# Infectious: Pathogens and How We Fight Them

**DOI:** 10.3201/eid2904.221820

**Published:** 2023-04

**Authors:** Nkuchia M. M’ikanatha

**Affiliations:** Pennsylvania Department of Health, Harrisburg, Pennsylvania, USA;; Pennsylvania State University College of Agriculture, University Park, Pennsylvania, USA

**Keywords:** Infectious, pathogens, book review

Infectious disease outbreaks can have profound societal ramifications (*1*), as underscored by the ongoing COVID-19 pandemic (*2*). *Infectious: Pathogens and How We Fight Them*, by John S. Tregoning, celebrates the research dedicated to understanding and controlling harmful microbes (Figure). An immunologist at Imperial College, London, Tregoning writes an accessible, authoritative primer, covering such topics as microbiology, epidemiology, and therapeutic solutions. He describes advances in techniques for identifying etiologic agents that have influenced scientific approaches to controlling, preventing, and even eliminating pathogens. He acknowledges pioneers such as Linnaeus and Gram and their work in categorizing organisms and differentiating bacteria (*3*). He also sheds light on the subject of pathogen–host interactions and genetic change: “Most mutations lead to nonsense… a minuscule number… [help] pathogens escape from the immune response and adapt to drugs.”

**Figure:**
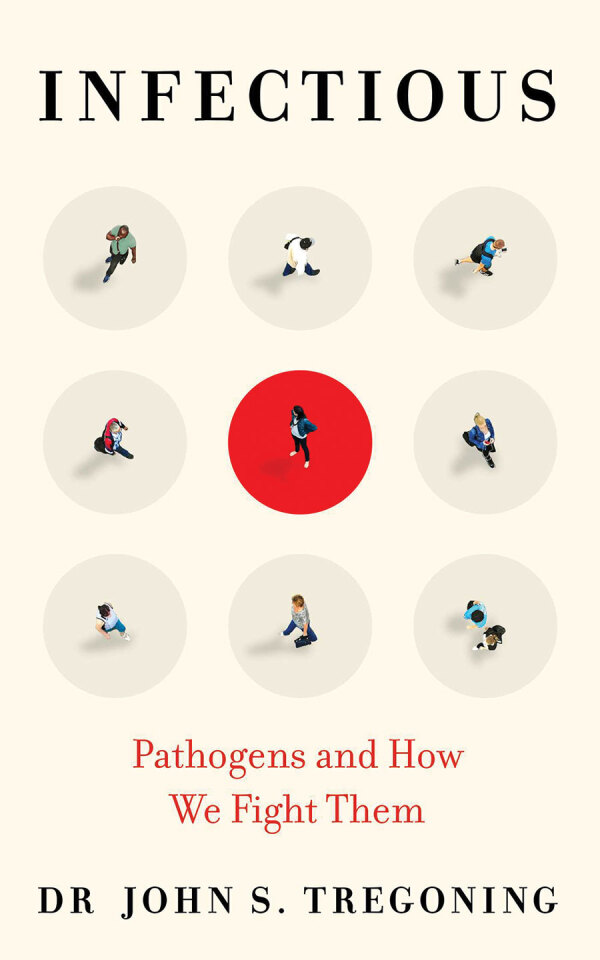
Infectious: Pathogens and How We Fight Them

The book provides readers a succinct description of innate and adaptive immunity, including how specific immune memory occurs when adaptive cells recognize, for example, a SARS-CoV-2 viral spike protein. Tregoning reviews how antibodies and T and B cells orchestrate immune responses and discusses how lifelong immunity can occur after recovery from certain diseases, such as measles. He shares a moving tribute to Brigitte Askonas for her contributions to our understanding of immune memory, made after she was forced to leave her homeland in 1938 because of her Jewish heritage. Tregoning also emphasizes that science thrives with diversity and inclusion, acknowledging trailblazers such as Alice Ball, an African American, for the first treatment of leprosy, and Tu Youyou, the Chinese Nobel laureate, for her discovery of artemisinin, an antimalarial drug. 

Tregoning convincingly explains why accurate diagnosis is crucial to epidemiologic investigations and therapeutic interventions. He provides examples of how causes of mysterious illnesses were determined, including *Chlamydia psittaci*–associated pneumonia among US sailors in 1929 and recent clusters of SARS-CoV-2 infections transmitted by asymptomatic persons. His optimistic assumption that we will continue to prevail against pathogenic microbes is bolstered by descriptions of breakthroughs in research on antimicrobials, smallpox, malaria, and COVID-19. He offers upbeat predictions of future advances, such as elucidation of the host-specific response to infections and using artificial intelligence to detect outbreaks. The author’s bright outlook is, nonetheless, tempered by recognition of undesirable consequences of inappropriate use of antimicrobial drugs.

Discussions of the threatening aspects of pathogens are countered by humorous observations and heartfelt vignettes. Tregoning jokingly describes Félix d’Hérelle’s “rather striking beard/moustache combo” in one moment and later describes crying uncontrollably after his son’s recovery from respiratory syncytial virus. Regarding how Barry Marshall identified the cause of gastric ulcers, he notes: “[He] proved the link by deliberately infecting himself with *Helicobacter pylori*, giving himself an ulcer and a Nobel Prize.” 

The book’s focus on “ologies” and therapeutics does not fully address social drivers of pathogens or grapple with barriers to science-based interventions (*5*). Tregoning also missed an opportunity to emphasize the need for strengthening the global mechanism for outbreak reporting and an enhanced integrated One Health approach to evolving threats (*6*,*7*).

*Infectious* will appeal to diverse audiences including biomedical trainees and policy makers because it transforms the discussion about harmful microbes into an engaging narrative. The book is an enjoyable and enlightening celebration of humanity’s achievements in the unending struggle with pathogens. Delivered with clarity, the book’s audio version is rewarding.
